# Comparative Transcriptome Analysis of *Bacillus subtilis* Responding to Dissolved Oxygen in Adenosine Fermentation

**DOI:** 10.1371/journal.pone.0020092

**Published:** 2011-05-18

**Authors:** Wen-Bang Yu, Shu-Hong Gao, Chun-Yun Yin, Ying Zhou, Bang-Ce Ye

**Affiliations:** Lab of Biosystems and Microanalysis, State Key Laboratory of Bioreactor Engineering, East China University of Science and Technology, Shanghai, China; Tel Aviv University, Israel

## Abstract

Dissolved oxygen (DO) is an important factor for adenosine fermentation. Our previous experiments have shown that low oxygen supply in the growth period was optimal for high adenosine yield. Herein, to better understand the link between oxygen supply and adenosine productivity in *B. subtilis* (ATCC21616), we sought to systematically explore the effect of DO on genetic regulation and metabolism through transcriptome analysis. The microarrays representing 4,106 genes were used to study temporal transcript profiles of *B. subtilis* fermentation in response to high oxygen supply (agitation 700 r/min) and low oxygen supply (agitation 450 r/min). The transcriptome data analysis revealed that low oxygen supply has three major effects on metabolism: enhance carbon metabolism (glucose metabolism, pyruvate metabolism and carbon overflow), inhibit degradation of nitrogen sources (glutamate family amino acids and xanthine) and purine synthesis. Inhibition of xanthine degradation was the reason that low oxygen supply enhanced adenosine production. These provide us with potential targets, which can be modified to achieve higher adenosine yield. Expression of genes involved in energy, cell type differentiation, protein synthesis was also influenced by oxygen supply. These results provided new insights into the relationship between oxygen supply and metabolism.

## Introduction

Adenosine plays an important role in biochemical and physiological processes including tissue protection and repair properties, neurotransmission and anti-inflammatory [1], [2]. Furthermore, it has important medical uses in heart diseases [3], as it plays a pivotal role in heart coronary circulation and heart protection and can also be used to effectively terminate certain supraventricular tachycardia (SVT) that involves atrioventricular (AV) node in the reentry pathway [4], [5].

Adenosine is produced mainly by industrial fermentation. It is known that the biosynthesis of nucleotide proceeds from 5′-phosphoribosyl-pyrophosphate (PRPP), which is formed from ribose-5′-phosphate and ATP. The first complete purine nucleotide, inosinic acid (IMP), is synthesized through several reactions, and then AMP is synthesized through branched pathways. Adenosine is finally synthesized by dephosphorylation of AMP [6]. Since 1968, Konishi *et al* have reported about adenosine fermentation [7]. Thenceforth, Haneda *et al* put great efforts to it [8], [9]. They found that a xanthine auxotroph *Bacillus* strain lacking adenase was a good adenosine producer; meanwhile excess guanine, a slightly acidic medium (pH 5.0–6.0) and sufficient oxygen supply were optimum conditions for adenosine biosynthesis.

Dissolved oxygen (DO) level is an important factor in aerobic fermentation that could significantly influence bacteria's metabolism and product yield. Oxygen plays an important role in biomass synthesis, cell morphology, biochemical degradation, electron transport and ATP availability [10], [11]. It has also been reported that oxygen can have an effect on various cell functions including carbon metabolism, antibiotic production and stress response [12]. The relationship between oxygen supply and fermentation productivity has been a focal point in aerobic fermentation and, thus optimization of DO concentration is always necessary for industrial bioprocess [13]–[17].

Our previous works also showed that oxygen supply is essential for adenosine-producing fermentation. However high-level of DO in the growth period restrains the overproduction of adenosine. The limitation of oxygen in the early stage is beneficial for the adenosine biosysthesis [18]. The intrinsic correlativity between oxygen supply and adenosine biosynthesis is still not well understood, and no study has attempted to comprehensively explore the mechanisms of how oxygen is integrated into the regulatory network of adenosine biosynthesis.

In this work we investigated the effect of the DO concentration on adenosine productivity by using comparative transcriptome analysis between adenosine fermentations with different oxygen supply. The results provided new insights to better elucidate the signaling network between DO level and adenosine yields, and provide guides for further improvement of adenosine production.

## Results

### Oxygen supply and adenosine yield

In this research, two batches of adenosine fermentation were conducted, one with high oxygen supply (agitation 700 r/min) and the other with low oxygen supply (agitation 450 r/min). As shown in Figure S1, in the early stage (0–20 h) the DO was at a very low level (almost zero, oxygen limitation) at 450 r/min agitation, while the DO was at a much higher level at 700 r/min agitation. The increasing DO after 20 h suggested that the oxygen supply was enough for fermentation in the later stage. Adenosine yield under low oxygen supply was twice (3.63 g/L) as under high oxygen supply (1.81 g/L). It was supposed that oxygen limitation in the early stage may contribute to a higher adenosine yield. Herein, transcriptome analysis was undertaken for *B. subtilis* to elucidate relationship between oxygen supply and adenosine yield.

### Functional category enrichment of transcriptome data

We studied the changes in gene expression in the early stage under high oxygen supply and low oxygen supply. Samples were taken respectively at 12 h and 18 h of fermentation process. Two independent cultured replicates were performed. The replicates were highly reproducible and the average coefficient of variation (CV) of the replicates was low (<16%). Pair-plots of intensities revealed a high Pearson correlation coefficient (>0.9, p<2.2e-16) (Text S1). Considering the samples from high oxygen supply as the control, we identified 434 (166 down-regulated genes, 268 up-regulated genes) genes at 12 h and 854 (424 down-regulated genes, 430 up-regulated genes) genes at 18 h as significantly differently expressed (more than two folds) genes (listed in Table S1 and Table S2). The transcriptome data were analyzed based on diverse sources of gene functions using two computational tools, including MIPS (http://mips.gsf.de/proj/biorel/bacillus_subtilis.html) and T-profiler analysis (http://www.science.uva.nl/~boorsma/t-profiler-bacillusnew/).

**Figure 1:**
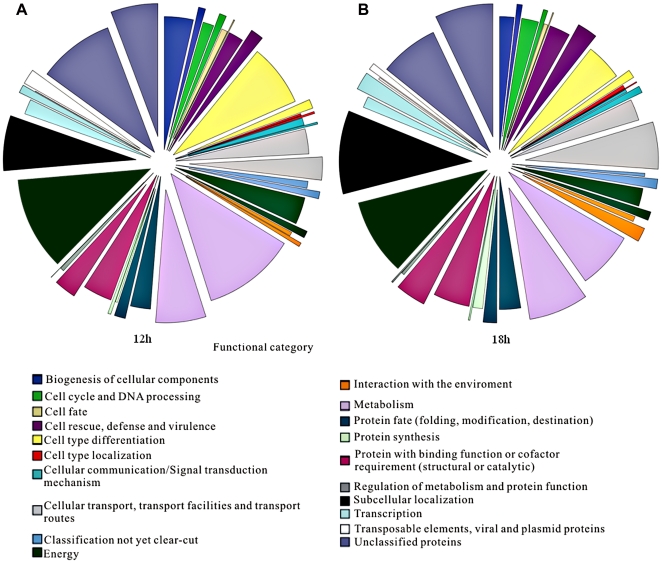
MIPS functional analysis of the differently expression genes (data from Table S1) at 12 h (A) and 18 h (B). The prominent part of the circle represents the down-regulated genes, and the other part represents the up-regulated genes.

MIPS functional analysis was used to assess functional category enrichments (Fig. 1 and Table S3). The up-regulated genes at 12 h exhibited a significant enrichment in functions of metabolism, energy, cell type differentiation and biogenesis of cellular components (Fig. 1A). Other significant categories in the up-regulated genes belong to the functions of cell cycle, protein fate, cell rescue and subcellular localization (Fig. 1A). The significant categories of the down-regulated genes at 12 h under low oxygen supply belong to the functions of metabolism and protein with binding functions (Fig. 1A). The up-regulated genes at 18 h showed significant enrichments in functions of energy, cell type differentiation and cell type localization (Fig. 1B). Other significant categories belong to the functions of metabolism, cell cycle, protein fate, protein with binding functions, cellular transport, cell rescue, biogenesis of cellular components and subcellular localization. The down-regulated genes at 18 h under low oxygen supply showed significant enrichment in cellular transport functions (Fig. 1B). Other significant categories belong to functions of metabolism, cell rescue, interaction with the environment and subcellular localization (Fig. 1B).

Transcriptional factors play a central role to restructure the transcriptome responses to environmental signals. The microarray data were subsequently analyzed using T-profiler to identify some transcriptional factors in response to DO level change. T-profiler is a computational tool that uses the *t*-test to score changes in the average activity of predefined groups of genes based on Gene Ontology categorization, upstream matches to a consensus transcription factor binding motif, or KEGG pathway [19]. It transforms transcriptional data of single genes into the behavior of gene groups, reflecting biological processes in cells (TF model, KEGG model and Subtilist model). In this study, all transcriptome data were online performed for T-Profiler analysis (http://www.science.uva.nl/~boorsma/t-profiler-bacillusnew/). The gene groups with significant T values (E-value, 0.05, TF model) are presented in Tables 1.

**Table 1:** Gene groups that with E-value<0.05 at 12 h or 18 h.

Time point of fermentation: 12 h					
Gene Groups	T-Value	E-value	Function	Significantly Regulated Genes (two-fold change)^a^	
SigE	28.91	<1.0e-15	Sporulation	*spoIIIAH,ywdL,spoIVA,spoVID,cotJC,cotJB,spoIIIAB,ysxE,spoIIIAC,safA,gerM,yngJ,spoIIIAF,spoIIIAA,ytrI,yloB,spoIIIAD,dacB,spoIIIAG,prkA,spoIIIAE,cotJA,ykvU,cwlJ,mmgA,spoVR,ydcC,cotE,glgA,glgD,spoIVFB,glgC,spmA,yngH,yhaX,ybaN,spoVB,yqxA,yqhV,mmgC,spoVD,yngG,spoIID,glgP,spoIIP,yngF,yhxC,yjbX,ylbO,yngE,glgB,spoIVFA,spoIIM,yqfD,yrrR,spoIIID,yndA,yngI,asnO,bofA,yxjC,yyaD,ytvI,ykvI,yxjF,scoA,usd,yesJ,ylbJ,mmgB,ypjB,yknT,yjbE,yhjR,scoB,ykvV,coxA,yabQ,yvjB,yjdH,ysnD,yjfA,spoVE*	
SpoIIID-Negative	15.29	<1.0e-15	Sporulation	*spoIIIAH,spoIVA,spoIIIAB,spoIIIAC,gerM,spoIIIAF,spoIIIAA,spoIIIAD,spoIIIAG,spoIIIAE,ykvU,spoIVFB,ybaN,spoVB,spoVD,spoIID,spoIVFA,yqfD,bofA,yitE,ylbJ,ypjB,ykvV,yabQ,spoVE*	
SigF	7.74	1.40E-11	Sporulation	*spoIIQ,lonB,dacF,ytfJ,yrrR,rsfA,csfB,ytfI,spoIIAB,spoIIR,katX,spoIIAA,ywhE,gpr,sigF,spoIVB,yhfM,yfhD,sspN,yhfW*	
ResD-Positive	7.62	3.50E-11	Respiration	*sboA,nasD,nasE,sboX,albA,albB,albC,resC,albF,resB,fnr,resD,resE,albD,albG*	
Rok-Nrgative	5.34	1.30E-04	Competence	*sboA,sboX,albA,albB,albC,comK,albF,albD,albG*	
Fnr-Positive	4.57	6.80E-03	Respiration	*ywcJ,narJ,narK,narH,narG,fnr,narI*	
SinR-Negative	-4.52	8.60E-03	Competence and Motility	*yvek,tasA,sipW,yvfA*	

Time point of fermentation: 18 h					
Gene Groups	T-Value	E-value	Function	Significantly Regulated Genes	
SigE	12.14	<1.0e-15	Sporulation	*spoIIIAH,spoIVCB,cwlJ,asnO,spoIIIC,yesJ,yheC,yngJ,spoIIID,ysxE,cotE,yjfA,yesK,cotJA,coxA,glgC,yknT,spoIIIAB,spoVK,spoVID,dacB,spoIIIAC,spoIID,prkA,usd,cotJB,yrrR,yngH,ydcC,spoIIIAG,yhaX,yrrS,spoIIIAD,yhxC,glgP,murB,spoIVFB,mbl,spoIIP,cotJC,glgA,spoIIIAA,ytvI,spoVR,yngG,gerM,bofA,ykvU,mmgA,bdbD,mmgC,spoIIIAF,yngE,ylbO,spoIVFA,spoIIM,spoVD,ypjB,spoIIIAE,spoIVA,ywdL,yndA,yqfD,yjdH,yjbE,yaaH,scoA,exuT,yunB,yxjC,ycgG*	
Strcon-Negative	6.34	3.20E-07		*atpA,rpmF,atpG,atpF,nusB,rplW,mbl,rpmC,tsf,rpsB,qoxB,rplQ,ylbN,ylaG,murE,rpsM,rplM,atpH,rpmD,rplO,rpmJ,pyrH,rpsL,rpsG,yrvE,dltA*	
SigF	5.89	5.40E-06	Sporulation	,*dacF,katX,sspN,spoIVB,rsfA,gpr,lonB,ytfJ,yrrR,yfhD,yrrS,spoIIQ,yqhP,yhfM,ytfI,csfB,yhcM,ylbC,gerAA,ywnJ,gerAC,gerAB*	
SpoIIID-Negative	5.34	1.30E-04	Sporulation	*spoIIIAH,spoIIIAB,spoIIIAC,spoIID,spoIIIAG,spoIIIAD,murB,spoIVFB,spoIIIA,gerM,bofA,ykvU,spoIIIAF,cotX,spoIVFA,spoVD,ypjB,spoIIIAE,spoIVA,yqfD,ycgG*	
SigB	5	8.00E-04	General Stress Response	*katX,ytxH,ytxG,ydaG,ysnF,dnaG,yacL,yfkI,ydaP,dps,yxkO,ctsR,sigA,yqhP,yocK,yvrE,yvgO,ydhK,clpP,yacK,yfhK,yfkJ,yhdO,yhaT,ydaS*	
ArfM-Positive	4.55	7.40E-03	Respiration	*alsS,alsD,nasD,nasE,dnaK,grpE,hemZ*	
Fur-Negative	-4.57	6.80E-03	Iron Metabolism	*dhbC,ydhU,ybbA,yclN,yfmD,dhbF,dhbA,dhbB,yusV,feuB,ydbN,dhbE,yfhA,nasE*	
CcpA-Negative	-5.96	3.50E-06	Carbon metabolism	*iolF,sigL,malP,galT,hutU,fbaB,treA,rbsR,licC,scoA,hutG,rbsB,pta,exuT,yfiA,treP,iolB,yxjC,glpF,rbsA,gntZ,iolC,iolH,bglS,malA,kdgA,rbsC,citM,rbsK,amyE,mmgA,mmgC,icd,glpK*	

The genes with two-fold change were analysed using T-Profiler and a table including gene groups with E-value<0.05 were generated automatically. Genes with two-fold change were listed in the table, and the down-regulated genes were underlined.

Seven co-regulated gene groups were found significantly disturbed by oxygen limitation at time point of 12 h in adenosine fermentation process, including SinR-Negative, FNR-Postive, Rok-Negative, ResD-Postive, SigF, SpoIIID-Negative, and SigE. Among them, five were related to sporulation and other cell fate (SinR, Rok, SigE, SigF, and SpoIIID); two were related to oxygen metabolism (FNR and ResD). In *B. subtilis*, SigE and SigF are both sporulation-specific sigma factors [20], while SpoIIID acts as a repressor during sporulation stage III to V [21], [22]. Significant positive T-Value of SigE, SigF and SpoIIID (Table 1) demonstrated that the genes dependent on SigE and SigF and genes repressed by SpoIIID were overexpressed. Those derepressed genes in the SpoIIID-Negative group are almost SigE-dependent (Table 1). SinR is a dual-functional regulator that activates motility and represses competence [23]. Negaive T-Value of SinR-Negative indicated that the genes negatively regulated by SinR were partially repressed. Indeed, genes including *yvfA*, *sipW*, *tasA*, and *yveK*, which encode biofilm matrix, were significantly repressed [24]–[27]. Rok is not only a repressor of competence, but also a repressor of a number of genes that encode products with antibiotic activity in *B. subtilis* [28]. Positive T-value of Rok-Negative revealed that the genes repressed by Rok regulator were derepressed. The significantly derepressed genes included *ablABCFDG-sboAX* and *comK* (Table 1). The *sbo-alb* operon was involved in subtilosin production [29] and was also directly positively regulated by ResD, an oxygen responser, which is activated by oxygen limitation [30].

FNR is, a sensor of oxygen that controls genes involved in facilitating adaptation to growth under oxygen limiting conditions, was induced by oxygen limitation [31]. Positive T-Value of FNR-Positive demonstrated that FNR-regulon was activated (Table 1). It was understandable that the genes in FNR-regulon were induced under low DO level. ResD is required for both anaerobic and aerobic growth [32]. When *B. subtilis* grows under oxygen limitation condition, it activates genes related to nitrate respiration. Positive T-Value of ResD-Positive showed that the genes of ResD-Postive group were activated, including *sbo-alb* operon, *nasDE*, *fnr*, and *resDE* (Table 1). The *nasDE* genes, members of *nasBCDEF* operon, which encode NADH-dependent nitrite reductase required for both anaerobic respiration and nitrogen metabolism, were induced by either nitrogen limitation or oxygen limitation [33], [34].

Eight significantly regulated gene groups were found at time point of 18 h in adenosine fermentation process, including SigE, Strcon-Negative, SigF, SpoIIID-Negative, SigB, ArfM-Positive, Fur-Negative, and CcpA-Negative (Table 1). ArfM, as a FNR-dependent regulator, is required for expression of *nasDE* and *hmp* [35]. T-profiler analysis showed a strong activation of genes in ArfM-regulon under low oxygen supply. ArfM and FNR are global transcriptional regulators that activate the expression of genes encoding many of the enzymes required for the anoxic environment. CcpA is a global regulator of carbon-metabolism in *B. subtilis* that controls carbon metabolism and mediates carbon metabolite repression (CCR) [36]–[38]. The significant negative T-Value for CcpA-Negative demonstrated that the genes of CcpA-Negative group were repressed, and indicated that oxygen limitation is beneficial for glucose metabolism in our experiment condition. SigB is a general stress respose regulator that controls at least 150 genes. The members of SigB-regulon are transiently induced following heat shock; salt, ethanol, or acid stress; or limitation of glucose and phosphate starvation [39]. In this study, we observed a significantly positive T-Value of SigB under low oxygen supply, which revealed that oxygen limitation activated the expression of some genes in SigB-regulon (Table 1). In *B. subtilis*, iron homeostasis is regulated by Fur, which represses expression of genes related to siderphore biosynthesis and iron uptake proteins [40]. Two factors have been proved to induce Fur-regulon: iron limitation and oxidative stress [41], [42]. The negative T-value of Fur-Negative showed that the gene group was repressed. Because culture medium used in our study was a rich medium, we hypothesized that the repression of Fur-regulon was likely related to oxidative stress, viz. high oxygen supply may result in oxidative stress to some degree. Strcon-Negative was involved in energy. Positive T-value indicated that genes of Strcon-Negative were partially derepressed. Obviously, functions of these gene groups are consistent with enriched functional categories.

### Effect of low oxygen supply on metabolism

#### Carbon metabolism

**Figure 2:**
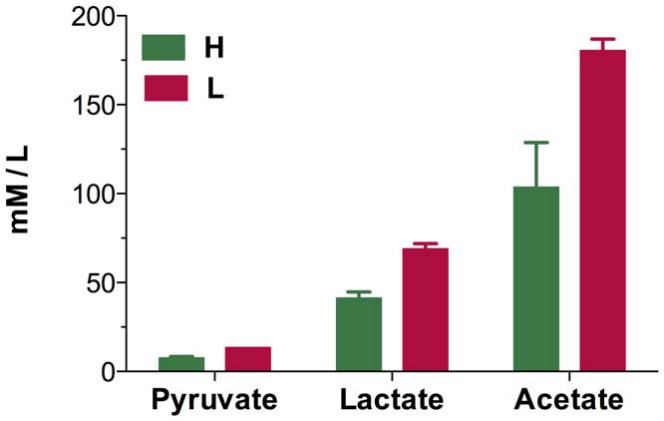
Accumulation of Pyruvate, Lactate and acetate at the final time of fermentation. L, Low oxygen supply; H, high oxygen supply.

The average consumption rate of glucose was much higher under low oxygen supply (0.64 g/(L·h)) than high oxygen supply (0.16 g/(L·h)). Consistent with the higher glucose consumption rate, a number of genes involved in glucose utilization were up-regulated under low oxygen supply. One glucose uptake gene *glcU*, whose expression was dependent on a forespore(late)-specific sigma factor (SigG) [43], was overexpressed at 18 h. Two genes involved in glycolysis (*gapA* and *eno*) were also up-regulated at 18 h. The pathways of pyruvate metabolism were promoted under the low oxygen supply. The *alsSD* operon (encoding acetolactate synthase and decarboxylase) was obviously up-regulated, suggesting that the acetoin formation from pyruvate, one of the overflow metabolism pathways that serve to excrete excess carbon from the cell, was activated by oxygen limitation. The operon *pdhABCD*, which encodes pyruvate dehydrogenase complex (PDH) during the biosynthesis of acetyl-CoA from pyruvate, was also induced under the low oxygen supply. Moreover, the genes *mmgABC* that involve in branched-chain amino acids and fatty acids degradation, which will generate additional acetyl-CoA, were up-regulated. Acetyl-CoA stands at crossroads of many metabolism pathways, such as citrate synthesis, acetate formation, fatty acid synthesis and amino acids synthesis (leucine, cysteine, methione, SAM, arginine and glycine). When grown under glucose-excess conditions, the *Bacillus subtilis* metabolizes a large proportion of the glucose only as far as pyruvate and acetyl-CoA and subsequently convert these compounds to by-products (lactate, acetate and acetoin) of metabolism, which are excreted to the extracellular environment [44]. Induction of *alsSD* and *pdhABCD* might suggest that the bacteria were in a glucose excess state under low oxygen supply. The genes involved in glycogen synthesis (*glgABCDP* at 12 h, *glgACP* at 18 h) were also up-regulated. These results were consistent with the study of Li *et al*, whose results also revealed that low agitation promoted glucose consumption rate in pyruvate fermentation of *Torulopsis glabrata* [45]. We also investigated accumulation of pyruvate, lactate, acetoin and acetate at the final time point of the fermentation processes. The results were well in agreement with transcriptome analysis. As shown in the Figure 2, concentrations of pyruvate, lactate and acetate, which are the metabolite products in carbon excess conditions, were much higher under low oxygen supply. However, the genes related to utilization of other carbon sources, such as *fruA*, *gamP*, *licC*, *malP*, *treP*, *iolBCFH*, *rbsACBK*, and *citM*, were all down-regulated by oxygen limitation. These genes were involved in the transport of fructose, glucosamine, lichenan, maltose, inositol, ribose and citrate respectively.

#### Metabolism of amino acids

**Figure 3:**
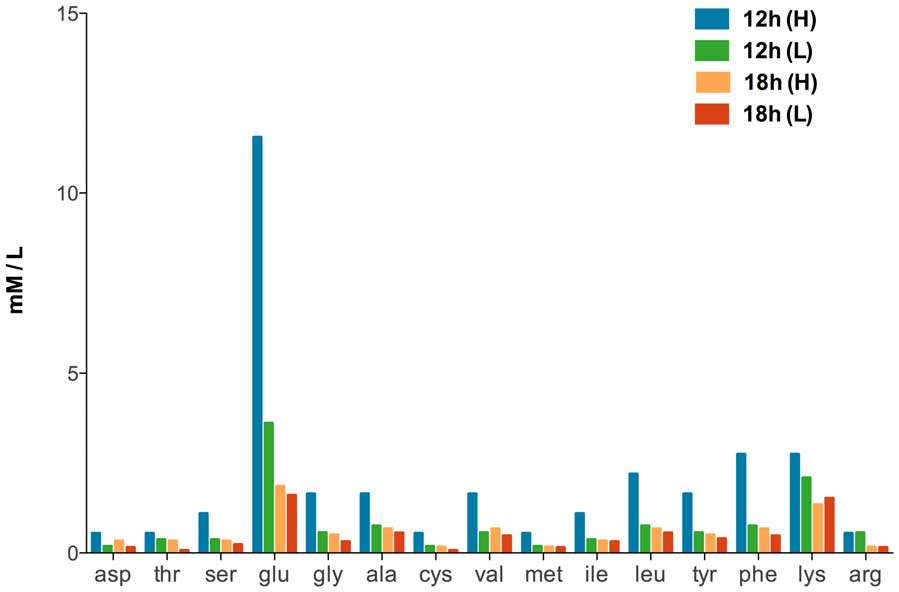
Intracellular amino acids concentration at 12 h and 18 h.

The intracellular amino acids in *B. subtilis* were analyzed. Most of the amino acids were in low concentrations except glutamate, which was most abundant in this study (Fig. 3). This was also found in other bacteria; for instance, in *B.megaterium* and *E. coli* the principle constituent of amino acids was glutamate [46], [47]. The intracellular pools of amino acids, especially glutamate, were more pronounced under high oxygen supply at 12 h in comparison to low oxygen supply, whereas the intracellular pools of amino acids were almost at the same level at 18 h (Fig. 3). Amino acids are important metabolites that must be maintained at an adequate level to serve for some physiology processes [48]. Lower concentration of amino acids under low oxygen supply indicated that the metabolic activity of amino acids was lower. This conclusion is supported by transcriptional observation.

In our transcriptome data, genes encoding glutamate family degradation pathways (*rocG*, *rocACDF* and *hutUG*) were down-regulated under low oxygen supply, while genes involved in glutamate synthesis (*gltAB*) were up-regulated. In bacteria, metabolism of glutamate modulates nitrogen-carbon metabolism balance and is well regulated through balancing distribution of 2-oxoglutarate. When the bacteria were in a carbon excess state, the 2-oxoglutarate will be siphoned off by glutamate synthase and catabolism of exiting amino acids will be avoided [44]. Up-regulation of *gltAB* and down-regulation of *rocG*, *rocAC*, and *hutUG* might indicate a carbon excess state the bacteria confronted under low oxygen supply. The concentration of 2-oxoglutarate was lower under low oxygen supply (data not shown). In microorganisms, 2-oxoglutarate always indicates nitrogen deficiency, while glutamine indicates nitrogen sufficiency [49]. Lower 2-oxoglutarate concentration might indicate that the nitrogen source was sufficient under low oxygen supply. This nitrogen sufficiency was probably resulted from enhanced glutamate synthesis. Genes involved in histidine synthesis (*hisA*, *hisFHIZ*), chorismate (*aroACF*) were repressed at either 18 h or 12 h under low oxygen supply. Because histidine and nucleotide synthesis uses the same precursor, phosphoribosyl pyrophosphate (PRPP), the decrease of histidine synthesis might prompt nucleotide synthesis. The genes of arginine synthesis pathway (*argBDFGH*) were up-regulated at 12 h, and the isoleucine synthesis pathway genes (*ilvABC*) were up-regulated at 18 h. Three genes (*bcd*, *bkdAA*, *bkdAB*) involved in degradation of branched-chain amino acids were up-regulated at 12 h under low oxygen supply. The *aspB* gene that involve in asparate synthesis was up-regulated at 18 h. Expression of genes encoding sulfur assimilation pathway (*ssuABC*) were significantly down-regulated at 18 h under low oxygen supply. A number of genes for ribosome assimilation (*rplMOQW*, *rpmCDFJ*, *rpoE*, *rpsABGLM*) were up-regulated at 18 h.

Low oxygen supply also exhibited great effect on the transport of amino acids. The glutamine ABC transporter genes (*glnHMPQ*) were significantly up-regulated under low oxygen supply, while ammonium uptake gene (*nrgA*) and proton/glutamate symport protein (glcT, glcP) were significantly down-regulated.

#### Metabolism of Purine Nucleotides

**Figure 4:**
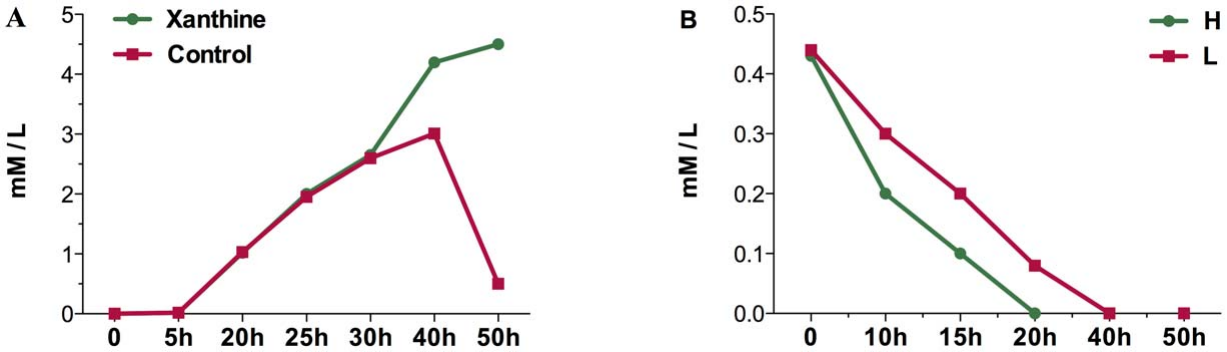
Effect of xanthine on adenosine production and concentration of xanthine over the time course of the fermentation process. (A) Effect of xanthine on adenosine production. The blue symbol denotes sufficient xanthine supply in the fermentation process. The red symbol denotes no xanthine addition in the fermentation process (B) Xanthine concentration over time course of the fermentation process under different DO levels.

The purine nucleotides metabolism that directly related to adenosine production was significantly influenced by oxygen supply. Eight genes of the xanthine catabolism (*pucH*, *pucM*, *puck*, *pucEDCBA*), which was inhibited by nitrogen sufficiency [50], were down-regulated under low oxygen supply. Since the adenosine producing strain was xanthine auxotroph, the repression of xanthine degradation probably benefits adenosine biosynthesis and growth. To confirm this hypothesis, we evaluated the effect of xanthine on adenosine production. It was found that adenosine production of the fermentation processes with sufficient xanthine was continuously and stable, whereas in the fermentation processes with no xanthine addition adenosine was degraded in the later period (Fig. 4A). This result strongly suggested that xanthine was important for adenosine production. The significance of the effect that xanthine exerted on adenosine production was tested using one-way ANOVA with Dunnett Mutiple Conparisions test (GraphPad InStat, GraphPad Software Inc., San Diego CA). The results showed appropriate xanthine addition could prompt adenosine production significantly (Text S2). Moreover, we analysed the xanthine concentration over the time course of fermentation process under different DO conditions. As shown in the Figure 4B, the xanthine of the medium was exhausted much earlier under high DO level than low DO level. These results further supported that inhibition of xanthine degradation was the reason that low oxygen supply enhanced adenosine production.

**Figure 5:**
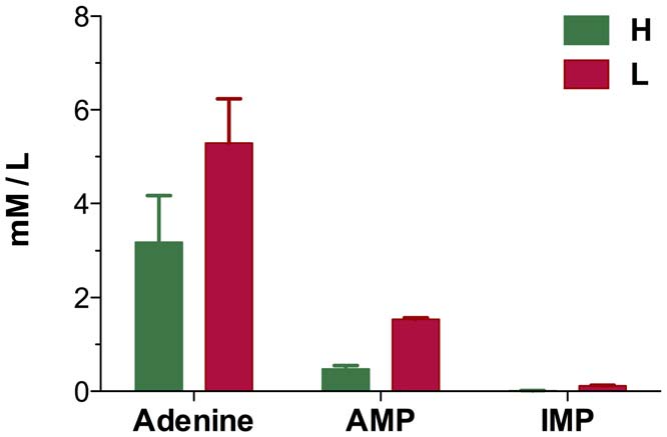
Accumulation of Adenine, AMP and IMP at the final time point of the fermentation process.

The *purA* and genes of *pur* operon (*purBCDEHKLMNQST*), which are directly involved in de novo purine nucleotide synthesis, were down-regulated under low oxygen supply at 12 h, whereas the genes of *pur* operon exhibited no significantly differential expression at 18 h except *purK* that was over expressed. Moreover, a gene (*pbuG*) involved in hypoxanthine/guanine permease was down-regulated. Obviously, down-expression of *pur* operon was a restriction for higher adenosine production under low oxygen supply. The purine synthesis pathway involved of a lot of substrates, such as glycine, PRPP, formate, glutamine, fumarate and asparate. In the transcriptome data, the genes related to glutamine transport (*glnHMPQ*) and asparate formation (*aspB*) were up-regulated. However, one gene (*gntZ*) of PRPP formation pathway was down-regulated at 18 h. The *pur* operon and *pbuG* were repressed by PurR whose activity was inhibited by PRPP and activated by adenine-contained compound. If the repression of *pur* operon and *pbuG* by PurR could be relieved, adenosine yield might be higher. Accumulation of other adenine nucleotides at the final time point of the fermentation process was also analyzed. The concentrations of adenine, AMP and IMP were also higher under low oxygen supply (Figure 5). These data suggested that low oxygen supply might benefits accumulation of adenine nucleotides. Higher accumulation of adenine nucleotide might explain why the *pur* regulon was repressed under low oxygen supply.

#### Coenzyme synthesis

Coenzymes are necessary small molecules that participate in enzyme-catalytic reactions. In the transcriptome-data, genes (*thiC*, *thiF*, *thiG*, *thiS*, *tenI*) encoding thiamine synthesis pathway and genes (*bioA*, *bioB*, *bioD*, *bioI*, *bioW*) encoding biotin synthesis pathway were up-regulated. Thiamine participates in the biosynthesis of acetyl-coA and pentose phosphate pathway (PPP) [51], while biotin was used in the glucogenesis. Enhancing of glucogenesis, has been proved to promote PPP pathway [52].

### Effect of low oxygen supply on respiration and sporulation

#### Respiration and energy metabolism

The oxygen limitation induced 14 bioenergetics-related genes, including 4 ATP synthase genes (*atpAFGH*) and 10 cytochrome oxidase genes (*qoxABCD*, *ctaCDEF* and *cydAB*). Induction of these genes showed that aerobic respiration and ATP synthesis were accelerated. Enhancement of ATP synthesis might reveal a demand for ATP or an energy starvation compared to the strain under high oxygen supply. It was also found the induction for *cydABCD*, *resABC*, and *qoxABCD* under anaerobic conditions [12]. The result may suggest that the induction of terminal oxidases may be needed to compensate for the lack of electron acceptors. Enhancement of ATP synthesis might reveal a demand for ATP or an energy starvation compared to the strain under high oxygen supply. Either demand for ATP or energy starvation would accelerate glucose consumption rate.

The *nasDE* and *narGHI* were all induced by low oxygen supply. It is known that *nasDE* genes encoding assimilatory nitrite reductases are induced by nitrogen limitation and oxygen limitation, while *narGHI* genes are induced by oxygen limitation only. Induction of *nasDE* and *narGHI* controlled by FNR showed that nitrite respiration is activated by low oxygen supply. Activation of aerobic respiration and nitrite respiration will accelerate NADH to be reoxidized to NAD^+^. Under low oxygen supply the glucose consumption rate was much higher; the bacteria may need more NAD^+^. Since the concentration of total NAD(H) is almost constant, the strain need to accelerate the regeneration rate of NAD^+^ from NADH.

#### Sporulation

From the transcription results, it can be seen that sporulation and competence were promoted under oxygen limitation. However, no spore formation was found in the fermentation progress (data not shown). It has been reported that D-ribose fermentation and riboflavin fermentation using the asporogenous mutant strain. It is suggested that sporulation relay may have impact on morphological phenotypes as well as metabolism. D-ribose production is related to cell elongation [53]. A random, transposon-tagged mutagenesis screening approach has found that some overproduction mutants were related to the cell morphological differentiation genes (*yloN*, *yjaU*, *phrC*, *cotE*, *sigW*, *fliP*) [54]. In our experiment, the glucose transport gene *glcU* (SigG dependant) was up-regulated, while the *phrC* and *cotE* are down-regulated. It is worth study whether there is any intrinsic relationship between the sporulation process and nucleotide substance (including adenosine) production.

### Oxygen limitation response network and adenosine production improvement

**Figure 6:**
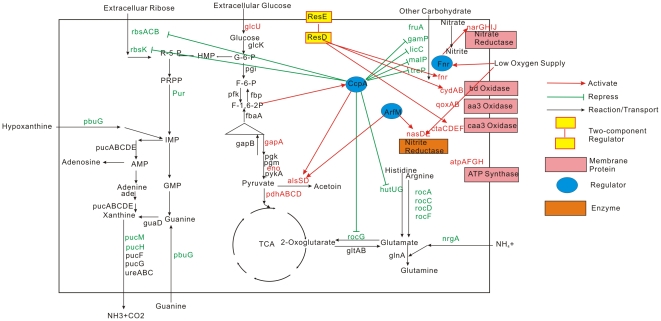
The putative signal network in response to low oxygen supply.

**Figure 7:**
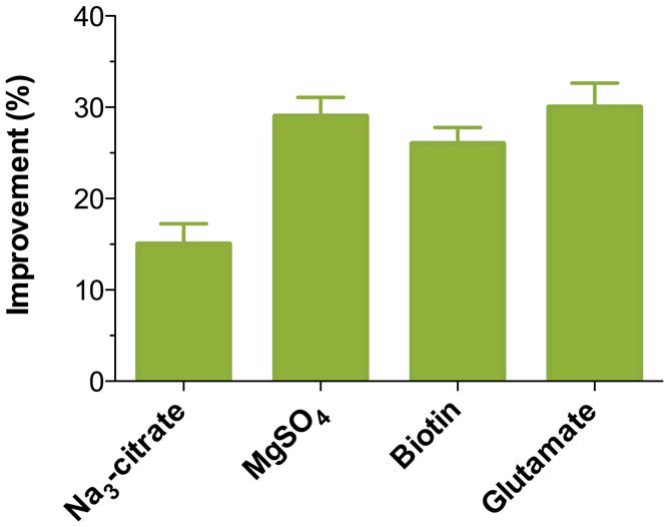
Effect of different addition on adenosine productivity.

Figure 6 illustrates the postulated response network associated with low oxygen supply. From the network, we can conclude that low oxygen supply enhanced glucose metabolism and carbon overflow. Degradation of glutamate family amino acids and xanthine were repressed by low oxygen supply. Purine nucleotide synthesis was inhibited by low oxygen supply. Low oxygen supply also induced respiration. Although the adenosine productivity was higher under low oxygen supply, there were two drawbacks in adenosine fermentation under low oxygen supply. Firstly, although glucose consumption rate was much higher under low oxygen supply, a part of the glucose consumed was used in the acetoin overflow pathway and the acetyl-CoA formation pathway. There was a hypothesis that inhibition of carbon overflow pathway may increase nucleotide production efficiency [55]. Chen *et al* reported that citrate is a good substrate for suppressing carbon overflow [55]. Herein, we investigated the effect of citrate on adenosine fermentation. As shown in the Figure 7, citrate could increase adenosine production by 15%. Secondly, the de novo purine nucleotide synthesis pathway was inhibited at 12 h. We supposed that, if the purine nucleotide pathway could be relieved from the repression, higher adenosine yield would be achieved. We also investigated the effect of Mg^2+^, thiamine and glutamate on adenosine yield. Mg^2+^ was co-transported with citrate in *B. subtilis*. Thiamine was involved glucogenesis, while glutamate was substrate for glucogenic conditions. Mg^2+^, thiamine and glutamate are all beneficial for adenosine yield (Fig. 7).

## Discussion

Although many studies have demonstrated that the dissolved oxygen level has great effect on the product yield or cell growth in fermentation, few of them were involved in the mechanism of these effects of DO on metabolism and genetic regulation. In this study, transcriptional profiling was used to assess how oxygen supply influences the metabolic and genetic regulatory network in adenosine-producing *B. subtilis*. The results revealed that oxygen supply significantly influenced regulatory works of SpoIIID-Negative, ResD-Positive Rok-Negative, FNR-Positive, SinR-Positive, Strcon-Negative, ArfM-Positive, Fur-Negative, and CcpA-Negative. SigB, SigE, and SigF-dependent transcription was significantly activated by low oxygen supply. Through transcriptome profiling analysis, we found that the limitation of oxygen significantly enhanced glucose utilization while slowing the catabolic rate of amino acids (glutamate, histidine and arginine). These results provide new insights for a better elucidation of the signaling networks between DO level and adenosine yield, and provide guides for setting up optimized oxygen supply strategies. This work is also a good reference for most aerobic *B. subtilis* fermentation for increasing metabolite productivity.

Inhibition of xanthine degradation is the reason that why low oxygen supply enhanced adenosine production. When grown under low oxygen supply, xanthine degradation was somehow inhibited. During the fermentation process, xanthine will be exhausted at the later period and adenosine production was inhibited. Inhibition of xanthine delayed the time point at which xanthine was exhausted, thus adenosine production was prolonged. However, why xanthine exhaustion limited adenosine yield is unknown. We hypothesized that the reason might be due to inhibition of adaptive reversion by xanthine. Since the producing strain (xanthine auxotroph) was selected by traditional method and no genetic manipulation was used, adaptive reversion of xanthine auxotroph that remove adenosine production capacity probably arise during adenosine fermentation. For nutrient auxotroph, nutrient sufficiency could inhibit adaptive reversion. Kodaira *et al* have reported that adaptive reversion of adenosine-producing strain was efficiently inhibited by guanine sufficiency [56]. As the xanthine degradation was inhibited under low oxygen supply, the time point at which xanthine was exhausted was probably delayed. So the adenosine-production period was prolonged. However, if xanthine could efficiently inhibit adaptive reversion need to be confirmed. Although the adenosine production was higher under low oxygen supply, enhancement of the carbon overflow pathway and inhibition of purine nucleotide synthesis were not conducive to adenosine production. Enhancement of carbon overflow pathway may probably reduce the carbon flow to PPP pathway, which generates ribose [57]. Herein, the citrate was used to inhibit carbon overflow and adenosine production was increased.

Under the limitation of oxygen, many genes coding for oxidases, nitrate reductase and nitrite reductase were up-regulated. These enzymes all were involved in recycle from NADH to NAD+. Since the concentration of NAD(H) is almost constant in the bacteria[58], turnover of NAD+ is very important for continuously NAD+ supplying for bacteria. Induction of these genes demonstrate that NAD+ availability was probably restricted, so the bacteria tried to accelerate NAD+ recycling. This restriction of NAD+ might be due to limited oxygen supply, as it was demonstrated that oxygen limitation will increase NADH level in *Bacillus subtilis* and activates Rex-repressed genes, which are activated by high ratio of NADH/NAD+ [59]. The *qoxABCD, ctaCDEF, cydAB,narGHI and nasDE* are all directly or indirectly positively regulated by ResDE (Dbtbs), which may sense the state of menaquinone [60]. Induction of these genes might demonstrate that ResDE was activated. Consistently, the T-Profiler also revealed that ResDE was activated. Activation of ResDE also demonstrated that the bacteria were probably in a reduced state compared to the bacteria under high oxygen supply. So we might conclude that oxygen limitation restrict NAD+ turnover rate and resulted in a reduced state. In a reduced state (NAD(P)H sufficient), bacteria will prefer pathways that involve dehydrogenases that utilize NAD(P)H to keep redox balance [61]. The reduced state under oxygen limitation will probably restrict TCA cycle, which is the major pathway that generates NADH, to keep redox balance. In *E. coli*, a reduced state (increased QH2/Q) will activate ArcAB and repress the TCA cycle [62], [63]. Although the genes in the TCA cycle was not repressed in this study, the genes *rocG,rocAC,hutU,hutG* which are involved in pathways that feed TCA cycle were all down-regulated. The acetoin-formation carbon overflow pathway, which was activated by glucose, was enhanced under low oxygen supply as *alsSD* were induced. Induction of carbon overflow pathway might refer to the reduced state of the bacteria grown under low oxygen supply. If the acetoin-formation overflow pathway was enhanced, carbon flow to TCA cycle might be reduced and additional NADH generation in the TCA cycle will be prevented.

## Materials and Methods

### Strains Fermentation

In this study, *Bacillus subtilis* ATCC 21616 (xanthine auxotrophic) was used. The starin was firstly grown on slant medium (10 g yeast extract, 10 ml corn syrup, 10 g peptone, 10 g agar powder per liter of distilled H_2_O; pH 7.0) for 48 h at 32°C. The strain was then transferred to seed medium (10 g yeast extract, 10 ml corn syrup, 10 g peptone, 10 g agar powder, 10 g sugar per liter of distilled H_2_O; pH 7.0), cultured on a shaker for 16 h at 32°C, 200 r/min. The seed was then inoculated into culture used for adenosine fermentation in a batch mode for 72 h. Two batches were conducted with two duplicate, one at high DO (agitation 700 r/min) and one at medium DO (agitation 450 r/min). The medium composition of the two batches was: 80 g glucose, 7.5 g yeast extract, 30 ml corn syrup, 10 g KH_2_PO_4_, 0.5 g MgSO_4_, 0.01 g MnSO_4_, 5 g NH_4_Cl, 10 g Monosodium glutamate (MSG) per liter distilled H_2_O, pH 7.5. The fermentation temperature was 32°C. Samples were respectively collected at 12 h and 18 h from two independent batches for transcriptome and metabolite analysis. Samples were quenched in liquid nitrogen immediately after collection, and then stored at −80°C.

### Analytical Method of Metabolites

Intracellular amino acids: 1.0 ml of *B. subtilis* culture was immediately centrifuged for 1 min at 10000 g at 4°C. Metabolites from the cells pellets were extracted with 200 µl 5% HClO_4_ in an ice bath for 15 min. After centrifugation at 10000 g for 5 min, the supernatant was neutralized with a K_2_CO_3_ solution, and the KClO_4_ precipitate was removed by centrifugation. The supernatant was stored at −20°C until use. The amino acids content was determined using an Amino acid Analyzer (L-8900 Hitachihitech, Japan) under the experimental conditions recommended for protein hydrolysates. Before analysis the sample was deproteinized by 10% Trichloroacetic acid (TCA) precipitation.

Organic acids and nucleoside: Organic acids and nucleoside were determined using a Waters HPLC system with Breeze Data Processor (Waters Corp. Milford, Massachusetts, USA). The separation was carried out on the Agilent Zorbax SB-Aq column (250 mm×4.6 mm, 5 µm) (Agilent, Palo Alto, CA, USA). A mobile phase of 0.01 mol/L H_2_SO_4_ solution (pH 2.0) was used at a flow rate of 0.6 mL/min. The column temperature was maintained at 30°C and the injection volume was 10 µl, the detection wavelength was 210 nm.

### Microarray construction and Hybridization

The *Bacillus subtilis* microarrays (BSU1.0) were customized using Agilent eArray program according to the manufacturer's recommendations. Each customized microarray (8×15K) contained spots in triplicate with 4,106 gene-specific 60-mer oligonucleotides representing the 4,106 protein-coding genes in *B. subtilis* (as reported for the *B. subtilis* genome at http://genolist.pasteur.fr/SubtiList/).

Samples stored at −80°C were used for RNA isolation. Total RNA samples were isolated from cultures by using Tiangen reagent according to the manufacturer's instructions (http://www.tiangen.com/newEbiz1/EbizPortalFG/portal/html/index.html). Then the RNAs were subsequently purified by QIGEN RNeasy Mini kit. The quality and quantity were determined by nanodrop UV spectroscopy and analyzed on a RNA 6000 Nano Labchip using 2100 bioanalyzer (Agilent technologies). Two milligrams RNA of each sample were used for cDNA synthesis. Then cRNA was subsequently synthesized using aaUTP. The amplified cRNA was purified using QIAGEN RNeasy Mini kit and the quality and quantity was determined using spectrophotometer at 260 nm and 280 nm. The purified cRNA was then labeled by Cy5 UTP. The fluorescently labeled cRNA was purified using QIAGEN RNeasy Mini kit. The purified fluorescently labeled cRNA was fragmentated in fragmentation buffer (Agilent). The prepared-as cRNA of 55 µl was mixed in 55 µl GE Hybridization Buffer HI-RPM (Agilent). Hybridization was performed in an Agilent Microarray Hybridization Chamber (G2534A) for 16 h at 65°C at rotation of 10 r/min. After the hybridization, the slides were washed in Gene Expression Wash Buffer (Agilent). Microarrays were scanned using an Agilent G2565BA scanner at 5 µm resolution. All data is MIAME compliant and that the raw data has been deposited in gene expression pmnibus (GEO) database.

### Microarray Data analysis

For data extraction, normalization and filtration, we used the methods as described in our previous work [19]. To identify differential expression genes responding to different DO, we used fold change method (2 fold as a cutoff value), considering the samples of high DO as control. Genes with fold change bigger than 2 were thought to be significantly disturbed genes responsive to oxygen supply. All differential expressing genes were uploaded for functional category analysis (http://mips.helmholtz-muenchen.de/proj/funcatDB/) with a threshold of 0.001 and T-profiler analysis (http://www.science.uva.nl/~boorsma/t-profiler-bacillusnew/) [19].

## Supporting Information

Figure S1 **DO and Adenosine Yield.** The DO during the fermentation was monitored and depicted. And the final adenosine yield was also showed.
(DOC)

Table S1 **Genes List of genes with two-fold change at 12 h.** All genes that changed more than two-fold at 12 h were listed. The change folds were also showed.
(XLS)

Table S2 **Genes List of genes with two-fold change at 18 h.** All genes that changed more than two-fold at 18 h were listed. The change folds were also showed.
(XLS)

Table S3 **MIPS functional analysis of the genes with two-fold change at 12 h and 18 h.** Genes that changed more than two-fold were analyzed for enrichment of functional category online (http://mips.gsf.de/proj/biorel/bacillus_subtilis.html).
(XLS)

Text S1 **Replicate and Reproducibility.** Reproducibility of replicate experiments was discussed.
(DOC)

Text S2 **The effect of xanthine addition on adenosine production.** One-way Analysis of Variance (ANOVA) was used.
(DOC)
