# Supplementary material for: Comparative Transcriptome Analysis of Bacillus subtilis Responding to Dissolved Oxygen in Adenosine Fermentation
Source: PLoS One. 2011 May 18;6(5):e20092. doi: 10.1371/journal.pone.0020092 (PMC3097244; doi:10.1371/journal.pone.0020092)
Supplement: Figure S1 — DO and Adenosine Yield. The DO during the fermentation was monitored and depicted. And the final adenosine yield was also showed. (DOC) [file pone.0020092.s001.doc]

**Supporting Information**

**Comparative transcriptome analysis of *Bacillus subtilis* responding to dissolved oxygen in adenosine fermentation**

**Wen-ang-Yu**, **Shu-Hong Gao**, **Bang-Ce Ye**^§^

Lab of Biosystems and Microanalysis, State Key Laboratory of Bioreactor, East China University of Science & Technology, Shanghai, China

Email addresses:

WBYu:wenbangyu123@126.com

SHGao:shhgao@ecust.edu.cn

BCYe:bcye@ecust.edu.cn


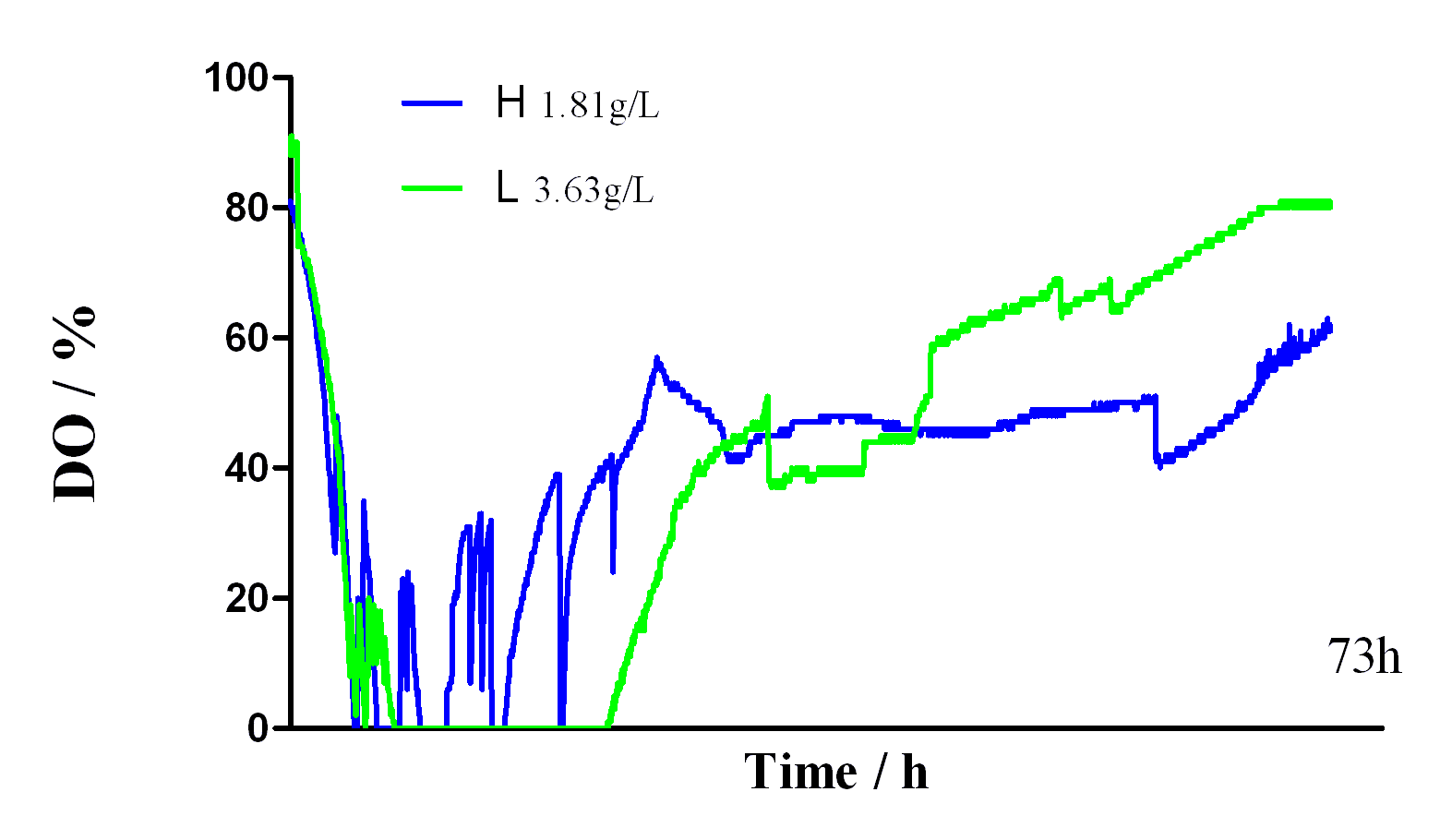


**Figure S1 - DO and Adenosine Yield**

Figure S1 Adenosine yield and Oxygen Supply. DO assay in the fermentation cultures during the process of fermentation with different oxygen supply. M represents low oxygen supply and H represents high oxygen supply.
