# Supplementary material for: Comparative Transcriptome Analysis of Bacillus subtilis Responding to Dissolved Oxygen in Adenosine Fermentation
Source: PLoS One. 2011 May 18;6(5):e20092. doi: 10.1371/journal.pone.0020092 (PMC3097244; doi:10.1371/journal.pone.0020092)
Supplement: Text S1 — Replicate and Reproducibility. Reproducibility of replicate experiments was discussed. (DOC) [file pone.0020092.s005.doc]

## Supporting Information:

## Comparative transcriptome analysis of Bacillus subtilis responding to dissolved oxygen in adenosine fermentation

**Text S1 Replicate and reproducibility**

Samples were taken at 12h and 18h. Two independent cultured replicates were performed. The log intensities demonstrated a high correlation between the two series of replicate mesurements. The average coefficient of variation (CV, S.D. divided by the mean) of the replicates at the two time points was below 16.5%:

The correlation values and average CV between biological replicates

| Time Points | Correlation value | p-value | CV(%) |
| --- | --- | --- | --- |
| 12h | 0.92 | <2.2e-16 | 16.42 |
| 18h | 0.95 | <2.2e-16 | 16.24 |
