# Supplementary material for: Comparative Transcriptome Analysis of Bacillus subtilis Responding to Dissolved Oxygen in Adenosine Fermentation
Source: PLoS One. 2011 May 18;6(5):e20092. doi: 10.1371/journal.pone.0020092 (PMC3097244; doi:10.1371/journal.pone.0020092)
Supplement: Text S2 — The effect of xanthine addition on adenosine production. One-way Analysis of Variance (ANOVA) was used. (DOC) [file pone.0020092.s006.doc]

We have examined the effect of xanthine addition on adenosine production using one-way ANOVA with Dunnett Mutiple Conparisions test (GraphPad InStat, GraphPad Software Inc., San Diego CA). Results in the table showed that appropriate xanthine addition could prompt adenosine production significantly at a level of p-value<0.01.

Table S1 Effect of Xanthine Addition on Adenosine Production

| xanthine addition, mg/L | adenosine production, g/L |
| --- | --- |
| 40 | 5.12±0.378** |
| 80 | 5.11±0.614** |
| 120 | 5.61±0.243** |
| 160 | 4.71±0.665^ns^ |
| 200 | 4.19±0.203^ns^ |
| control | 3.66±0.331 |

Table S2 Dunnett Multiple Comparisons Test*. Data are expressed as mean values ± SD (n = 3) ** *p*-value< 0.01. ns :not significant, p-value>0.05

| Comparison | Mean Difference | q | P value |
| --- | --- | --- | --- |
| control vs xan-40 | -1.460 | 4.040 ** | P<0.01 |
| control vs xan-80 | -1.450 | 4.013 ** | P<0.01 |
| control vs xan-120 | -1.947 | 5.387 ** | P<0.01 |
| control vs xan-160 | -1.047 | 2.897 ns | P>0.05 |
| control vs xan-200 | -0.5267 | 1.457 ns | P>0.05 |

*If the value of q is greater than 2.900 then the P value is less than 0.05.
